# Long non-coding RNA Lnc-Tim3 exacerbates CD8 T cell exhaustion via binding to Tim-3 and inducing nuclear translocation of Bat3 in HCC

**DOI:** 10.1038/s41419-018-0528-7

**Published:** 2018-04-30

**Authors:** Jie Ji, Yin Yin, Huanyu Ju, Xiaoliang Xu, Wei Liu, Qiang Fu, Jiaojiao Hu, Xudong Zhang, Beicheng Sun

**Affiliations:** 1 Liver Transplantation Center of the First Affiliated Hospital and State Key Laboratory of Reproductive Medicine, Nanjing Medical University, Nanjing, Jiangsu Province China; 2 Department of Laboratory Medicine, the First Affiliated Hospital of Nanjing Medical University, Nanjing, Jiangsu Province China; 3 Department of Gastroenterology, The Second Affiliated Hospital of Nanjing Medical University, Nanjing, Jiangsu Province China; 4 Department of General Surgery, The Affiliated Changzhou No.2 People’s Hospital of Nanjing Medical University, Nanjing, Jiangsu Province China

## Abstract

Although one of the first comprehensive examinations of long non-coding RNA (lncRNA) expression was performed in human CD8 T lymphocytes, little is known about their roles in CD8 T cells functions during the progression of hepatocellular carcinoma (HCC). Here, we show that Lnc-Tim3 is upregulated and negatively correlates with IFN-γ and IL-2 production in tumor-infiltrating CD8 T cells of HCC patients. Lnc-Tim3 plays a pivotal role in stimulating CD8 T exhaustion and the survival of the exhausted CD8 T cells. Mechanistically, Lnc-Tim3 specifically binds to Tim-3 and blocks its interaction with Bat3, thus suppressing downstream Lck/ NFAT1/AP-1 signaling, leading to nuclear localization of Bat3, and enhancing p300-dependent p53 and RelA transcriptional activation of anti-apoptosis genes including MDM2 and Bcl-2. In summary, Lnc-Tim3 promotes T cell exhaustion, a phenotype which is correlated with compromised anti-tumor immunity, suggesting that Lnc-Tim3 and its associated signaling pathways may influence the outcome of cancer therapies aimed at modulating the acquired immune system.

## Introduction

Hepatocellular carcinoma (HCC) is an inflammation-related cancer and the third leading cause of cancer-related death worldwide^1^. It is known that persistent inflammation exacerbates HCC development^2^. The evidence demonstrates that immune checkpoint molecules play an important role in immune evasion of HCC. Immunological studies are revolutionizing HCC immunotherapy^3^. The presence of tumor-infiltrating lymphocytes (TILs) is responsible for HCC immunogenicity^4^. Generally, most tumor cells express antigens that can be recognized by CD8 T cells, which trigger antitumor immune responses. These tumor-associated antigen (TAA)-specific CD8 T-cell responses positively influence the survival of HCC. The TAA-specific cytotoxic CD8 T cells are the key players in most immunotherapy studies in HCC^5^. However, TAAs-specific CD8 lymphocytes from TILs produce less IFN-γ than ones in peripheral blood, indicating the CD8 T cells display exhaustion in tumor microenvironment^4,6^. Accordingly, it has been proposed that an overcoming of immunosuppressive intratumor environment might potentially restore successful antitumor immunity.

Immune checkpoint molecules contribute to HCC immunosuppressive through suppressing the anti-tumor immune response^7^. T cell immunoglobulin mucin 3 (Tim-3, HAVCR2, Gene ID: 84868, located in chromosome 5), a member of immune checkpoint proteins, acts as an inhibitory receptor for T cells^8^. The interaction of Tim-3 with its ligand, galectin-9 (Gal-9), induces cell death. Tim-3 has been found in differentiated IFN-γ-producing CD4^+^ T helper type 1 and CD8^+^ T cytotoxic type 1 cells^9^. It has been reported that Tim-3 is mostly expressed on CD8 TILs of solid tumor^10^. However, Tim-3 does not contain any obvious inhibitory signaling motifs and leads to augmentation of T-cell receptor (TCR)-dependent signaling pathways in T cells. Moreover, the activating of Tim-3 can convey a death signal into T cells. How then do Tim-3^+^ exhausted CD8 T cells persist in HCC TILs?

More evidence shows that long non-coding RNAs (lncRNAs) regulate a diversity of biological functions. In the field of immunology, recent studies have shown extensive changes in lncRNAs expression during T cell development, differentiation, and activation^11^. The majority of the lncRNAs are expressed in a stage-or lineage-specific manner, however just few mRNAs display this property^12^. These facts suggest that T cell-specific lncRNAs play a vital role in the complexity of the T cell compartment^13^. For example, NeST is expressed in Th1 CD4 T cells, CD8 T cells, and natural killer cells. The nucleus-located NeST interacts with WDR5 and induces the expression of IFN-γ in activated CD8 T^14^. However, further efforts are needed to demonstrate whether lncRNAs exert their biological functions in T cells of tumor microenvironment.

In our previous studies, high-throughput screening has been used to explore the transcriptomic associations between lncRNAs and mRNAs in the TILs of HCC patients. In this study, the expression of Lnc-Tim3 (ENST00000443947.1, AC011288.2, located in chromosome 7) was upregulated in CD8 T cells from HCC TILs. Lnc-Tim3 correlates with the exhaustion of CD8 T lymphocytes and the correlated mechanisms are studied. The results indicate that Lnc-Tim3 binds to Tim-3 and leads to release of Bat3, thereby reducing the stimulation of Lck and its downstream AP-1/NFAT1 signaling. However, Lnc-Tim3 protects from Gal-9-mediated cell death. The results show that released Bat3 enhances the recruitment of p53 and RelA to p300 and facilitates subsequent transcription of anti-apoptotic genes. Altogether, Lnc-Tim3 promotes CD8 T cell exhaustion and survival, a phenotype which is correlated with compromised anti-tumor immunity.

## Results

### Upregulated Lnc-Tim3 correlates with the exhaustion of CD8 T lymphocytes

**Fig. 1:**
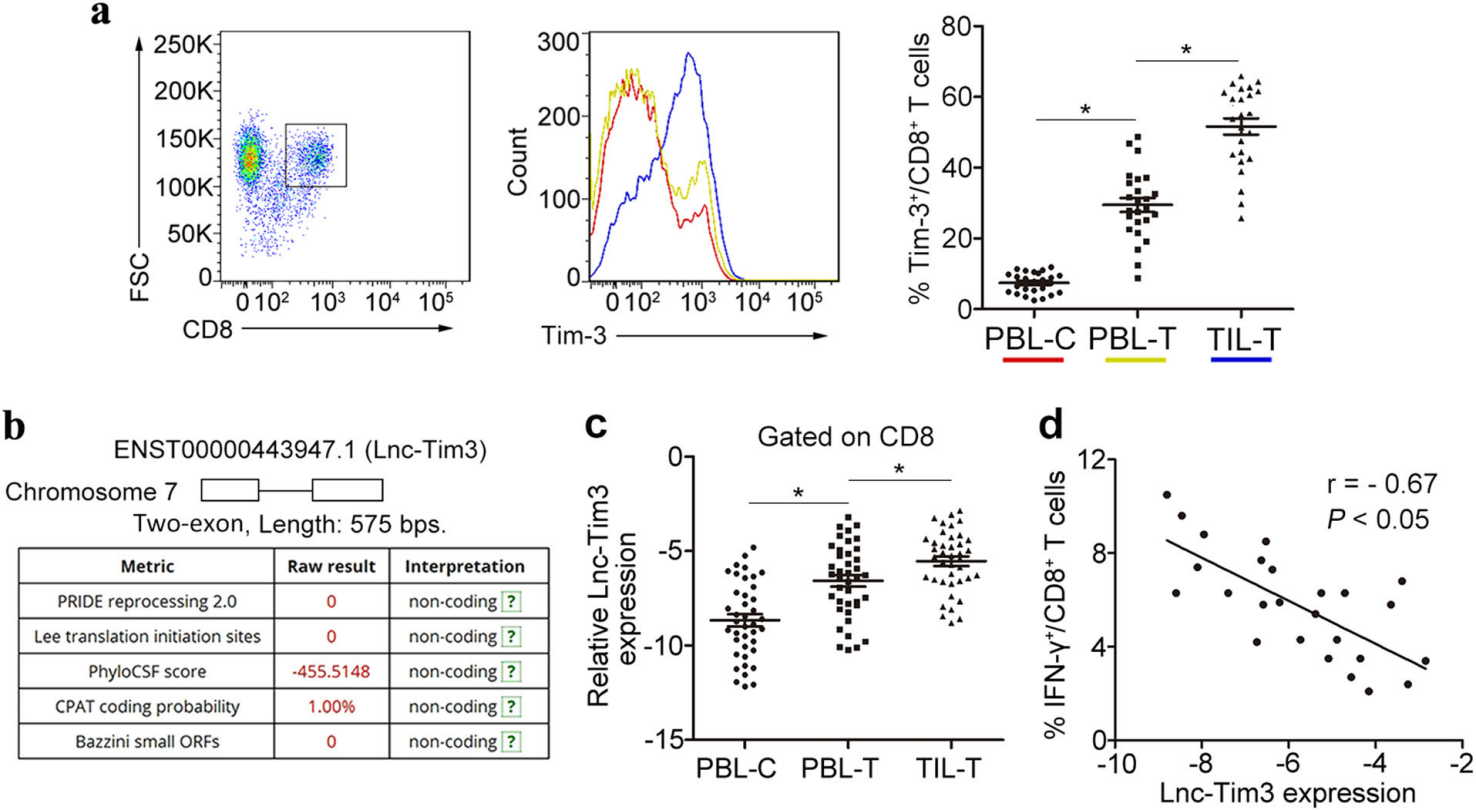
Upregulated Lnc-Tim3 correlates with the exhaustion of CD8 T lymphocytes. **a** The percentage of Tim-3^+^ CD8 T cells in the infiltrated CD8 T cells was determined by flow cytometry. The percentages of Tim-3^+^ CD8 T cells in the tumor-infiltrating CD8 T cells (TIL-T) and peripheral blood CD8 T cells of HCC patients (PBL-T) and healthy controls (PBL-C) was analyzed. *n* = 25 for each group. **b** The protein-coding potential and the ORF size (a two-exon gene) of long non-coding RNA Lnc-Tim3 (ENST00000443947.1) were analyzed by databases. **c** Relatively increased levels of Lnc-Tim3 were confirmed in tumor-infiltrating CD8 T cells (TIL-T) by comparing them with those in the peripheral blood CD8 T cells of HCC patients (PBL-T) and healthy controls (PBL-C). *n* = 40 for each group. **d** The Pearson’s correlation of Lnc-Tim3 expression and the percentage of IFN-γ^+^ CD8 T cells in the tumor-infiltrating CD8 T cells was analyzed. (*n* = 25). Data are presented as means ± S.E.M. (*P < 0.05)

Tim-3 has been shown to negatively regulate T-cell-dependent immune responses and was recently demonstrated to be associated with the phenomenon of immune exhaustion^15^. Others have reported that Tim-3 is mainly expressed on CD8 TILs in mice bearing solid tumors and human cytotoxic T type 1 (T_C_1) CD8 cells^16^. Tim-3^+^ TILs exhibit the most severe exhausted phenotype as defined by failure to proliferate and produce IL-2, TNF, and IFN-γ^10^. To examine a potential role for Tim-3 in T cell exhaustion in HCC, we first examined the expression of Tim-3 in CD8 T cells via flow cytometry analysis. We found that the percentages of Tim-3^+^ CD8 T cells was highly upregulated in tumor-infiltrating T cells compared to the peripheral blood T cells from HCC patients and healthy controls (Fig. 1a). In our previous study, transcriptome profiling of lncRNA-mRNA co-expression networks comparison between HCC TILs and peripheral blood lymphocytes (PBLs) have been done^17^. In the present study, we mainly focused on the dysregulated lncRNAs and related lncRNA-mRNA co-expression networks in tumor-infiltrating CD8 T cells. According to our previous study, we found Tim-3 and Lnc-Tim3 (ENST00000443947.1) showed the greatest difference. Both the mRNA and lncRNA screening indicated that the different expression of the candidate RNAs mainly occurred in CD8 T cells, rather than CD4 T cells. To ensure that the novel candidates that we predicted did not encode proteins, we used GeneID and CPAT to measure the protein-coding potential and the ORF size in Lnc-Tim3 sequence (Fig. 1b). To further understand the functions of the Lnc-Tim3, we first conducted real-time PCR assay to confirm its abnormal expression in the CD8 T cells from 40 individuals with HCC and 40 healthy controls. We found that Lnc-Tim3 was highly upregulated in tumor-infiltrating T cells compared to the peripheral blood T cells from HCC patients and healthy controls (Fig. 1c). Moreover, further flow cytometry analysis confirmed that a negative correlation was identified between Lnc-Tim3 expression and the percentage of IFN-γ^+^ CD8 T cells in the tumor-infiltrating CD8 T cells of HCC patients (Fig. 1d), suggesting that the presence of Lnc-Tim3 is associated with the exhaustion of CD8 T lymphocytes.

### Lnc-Tim3 specifically binds to the intracellular (IC) domain of Tim-3

**Fig. 2:**
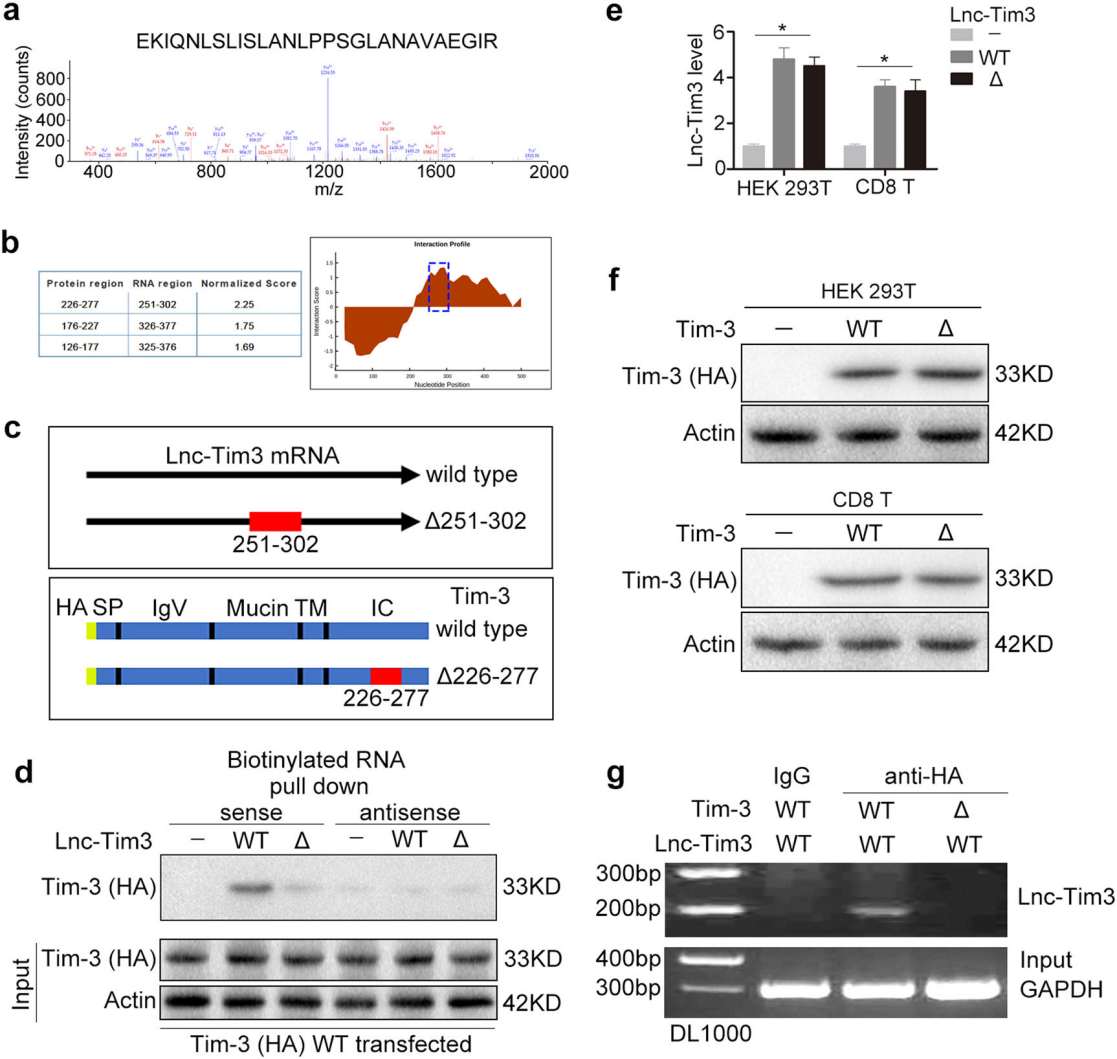
Cytoplasmic Lnc-Tim3 specifically binds to Tim-3. **a** Lnc-Tim3 RNA pull-down assay was performed. The associated proteins were processed and subjected to mass spectrometry followed by analysis via the Proteome Discoverer program. **b** The interaction between Tim-3 and Lnc-Tim3 was predicted by catRAPID method. **c** A schematic map of potential Tim-3 binding regions (wild type and Δ251-302) in the intracellular domain of Lnc-Tim3 (wild type and Δ226-277). **d** HEK 293 T cells were transfected with wild type Tim-3-HA (wild type) plasmid followed by lysis. Biotin-labeled sense and antisense of Lnc-Tim3 (wild type and Δ226-277) were used as probe. RNA pull-down assay was performed and the associated proteins were detected with anti-HA antibody. Representative of three experiments. **e** HEK 293 T and CD8 T cells were transfected with indicated lentiviral particles (vector, wild type or Δ226-277 Lnc-Tim3). The mRNA levels of Lnc-Tim3 were assessed by real-time PCR at 24 h after transfection. Data are presented as means ± S.E.M. *n* = 3, *P < 0.05. **f** HEK 293 T and CD8 T cells were transfected with indicated lentiviral particles (vector, wild type or Δ251-302 Tim-3-HA). The expression of Tim-3 (HA-tag) was assessed by western blot at 24 h after transfection. Representative of three experiments. **g** RIP assays were performed using HEK 293 T cells transfected with Tim-3-HA (wild type or Δ251-302) and Lnc-Tim3 (wild type), anti-HA antibody. The precipitated RNAs were determined by PCR for Lnc-Tim3 and GAPDH. Representative of three experiments

We next used a RNA pull-down assay with biotinylated Lnc-Tim3 to search for potential Lnc-Tim3-interacting proteins. Mass spec analysis of pull-down proteins revealed that the previously predicted Lnc-Tim3 partner, Tim-3, was a Lnc-Tim3-associated protein (Fig. 2a). On the basis of these results, the bioinformatic software catRAPID was used to predict the potential Tim-3 binding regions in Lnc-Tim3 and the potential protein-binding domains for Lnc-Tim3 in Tim-3 (Fig. 2b). As shown in Fig. 2b, c, the predicted region (nucleotides 251 to 302) in the Lnc-Tim3 were predicted to bind to Tim-3 and the amino acids between 226 and 277 in the IC domain of Tim-3 were suggested to be the most possible Lnc-Tim3 binding domain. Our pull-down results showed that deletions in the 251 to 302 regions of Lnc-Tim3 abrogated its binding ability to Tim-3 (Fig. 2d). Additionally, the deletion of the Lnc-Tim3 binding region (nucleotides 251 to 302) or Tim-3 binding region (amino acids 226 to 277) did not affect the levels of Lnc-Tim3 or Tim-3 protein, respectively (Fig. 2e,f). The results suggested that deletion mutant of Lnc-Tim3 and Tim-3 did not affect their stability. Furthermore, RNA immunoprecipitation (RIP) assays revealed that only anti-HA (wild type Tim-3) antibodies specifically precipitated Lnc-Tim3, but deletions in the amino acids 226 to 277 of Tim-3 abrogated its binding ability to Lnc-Tim3 (Fig. 2g). Taken together, our results suggest that Lnc-Tim3 specifically interacts with Tim-3 and that the predicted region (251 to 302) of Lnc-Tim3 is important for Lnc-Tim3 to bind to Tim-3.

### Lnc-Tim3 promotes CD8 T cell exhaustion but protects from galectin-9-mediated cell death

**Fig. 3:**
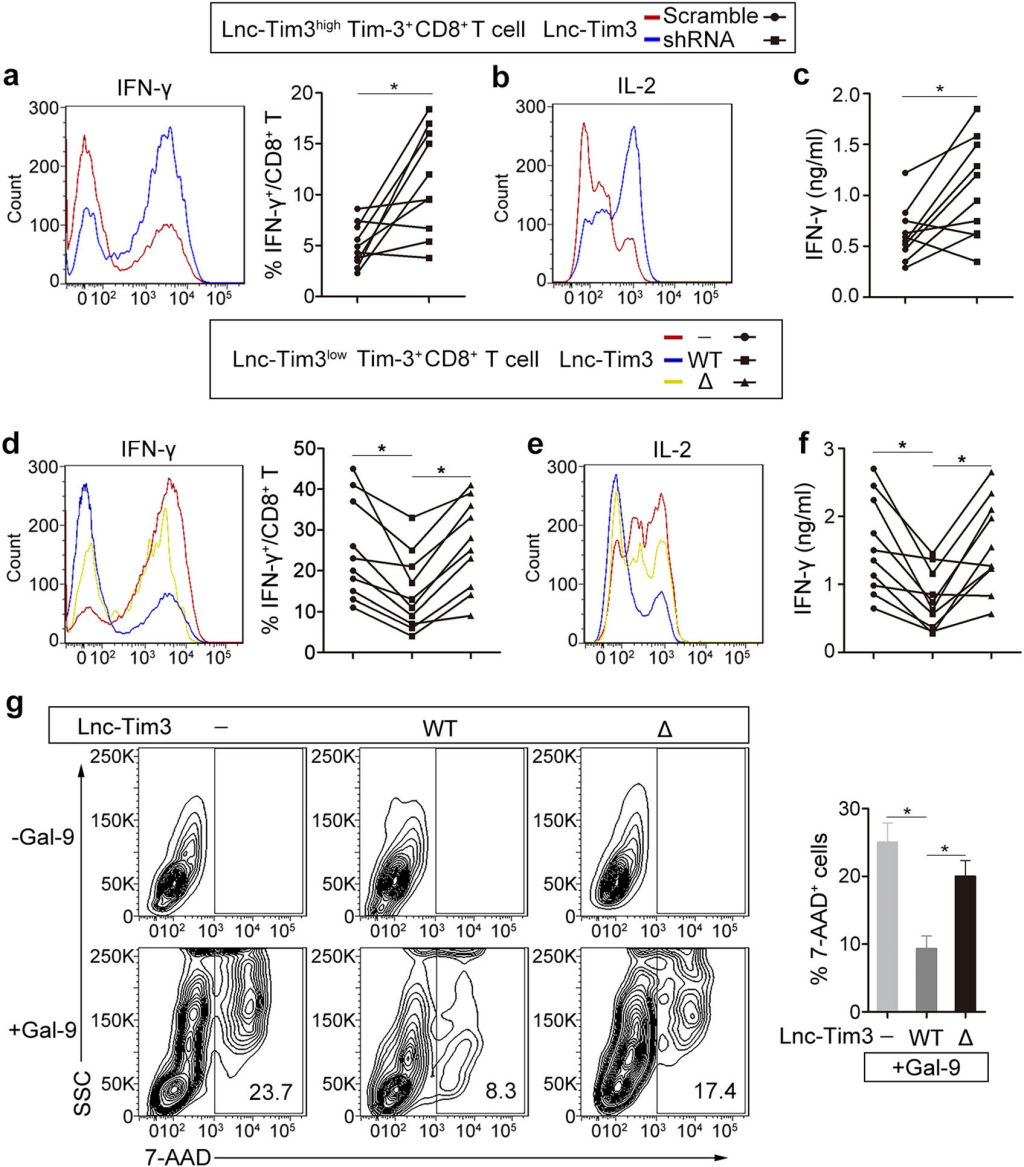
Lnc-Tim3 suppresses CD8 T cell function and protects from galectin-9–mediated cell death. **a**,** b** Tim-3^+^CD8 T cells isolated from tumor-infiltrating lymphocytes (TILs) of HCC patients with Lnc-Tim3 high expression (Lnc-Tim3^high^Tim-3^+^CD8 T; cut-off, -6.53) were transfected with indicated lentiviral particles (Lnc-Tim3 scramble or shRNA). Intracellular expression of IFN-γ (**a**, left) and IL-2 (**b**) in CD8 T cells after treated with anti-TCR/anti-CD28 beads (bead-to-cell ratio of 1:1), IL-12 (100 ng/ml), and peptide cocktail (AFP_158-166_, GPC_3144-152_, and NY-ESO-1_157-165_) were assessed by flow cytometry. The percentage of IFN-γ^+^CD8 T cells was showed in the right panel. (*n* = 10). **c** Concentration of IFN-γ in culture supernatant 48 h of the above groups. (*n* = 10). **d**,** e** Tim-3^+^CD8 T cells isolated from TILs of HCC patients with Lnc-Tim3 low expression (Lnc-Tim3^low^Tim-3^+^CD8 T; cut-off, −6.53) were transfected with indicated lentiviral particles (Lnc-Tim3 wild type or Δ226-277). Intracellular expression of IFN-γ (**d**, left) and IL-2 (**e**) in CD8 T cells after treated with anti-TCR/anti-CD28 beads (bead-to-cell ratio of 1:1), IL-12 (100 ng/ml), and peptide cocktail (AFP_158-166_, GPC_3144-152_, and NY-ESO-1_157-165_) were assessed by flow cytometry. The percentage of IFN-γ^+^CD8 T cells was showed in the right panel. (*n* = 10). **f** Concentration of IFN-γ in culture supernatant 48 h of the above groups. (*n* = 10). **g** Jurkat T cells were transfected with indicated lentiviral particles (Tim-3–HA, Lnc-Tim3 wild type or Δ226-277). Frequency of apoptosis were assessed by 7-aminoactinomycin D (7-AAD) after cells treated with galectin-9 for 3 h. Numbers contained in the fluorescence-activated cell sorting (FACS) plots represent the frequency of 7-AAD^+^ cells. SSC, side scatter. Data in the bar graph (right) represent the percentage of 7-AAD^+^ cells after galectin-9 treatment divided by the percentage of 7-AAD^+^ cells before treatment. Data are presented as means ± S.E.M. (*P < 0.05)

In other studies, the IC domain is required for Tim-3 signaling to T-cell activation pathways in a redundant fashion^15^. To further understand the functions of the Lnc-Tim3, Tim-3^+^CD8 T cells were sorted by flow cytometry from TILs of HCC patients. Subsequently, a series of in vitro experiments were performed to investigate the pro-exhaustion effects of Lnc-Tim3 in CD8 T cells. Silencing of endogenous Lnc-Tim3 by a lentivirus expressing Lnc-Tim3-targeting shRNA was employed in the Lnc-Tim3^high^Tim-3^+^CD8 T from TILs, and we found that Lnc-Tim3-sliencing cells produced greater amounts of IFN-γ (Fig. 3a, left) and interleukin-2 (IL-2) (Fig. 3b) than control cells after treatment with anti-TCR/anti-CD28 beads, interleukin-12 (IL-12), and peptide cocktail. Additionally, Silencing of Lnc-Tim3 resulted in increasing the percentage of IFN-γ secreting CD8 T cells (Fig. 3a, right) and significantly more IFN-γ secreting (Fig. 3c). Furthermore, we lentivirally overexpressed wild type and mutant (Δ251-302) forms of Lnc-Tim3-HA in Lnc-Tim3^low^Tim-3^+^CD8 T from TILs, and found that Lnc-Tim3-WT, but not Lnc-Tim3-Δ, strikingly reduced the amounts of IFN-γ (Fig. 3d, left) and IL-2 (Fig. 3e), the percentage of IFN-γ secreting CD8 T cells (Fig. 3d, right) and IFN-γ secretion (Fig. 3f) after treatment with anti-TCR/anti-CD28 beads, interleukin-12 (IL-12), and peptide cocktail. It is known that triggering of Tim-3 can transmit a death signal into T cells^18^. To directly assess the effect of Lnc-Tim3 on signaling through the galectin-9–Tim-3 axis, we assessed the ability of galectin-9 to induce the death of Lnc-Tim3 (wild type and mutant)-overexpressing Jurkat T cells. Overexpression of Lnc-Tim3_WT_, but not Lnc-Tim3_Δ251-302_, showed a lower percentage of Tim-3-transfected Jurkat T cells underwent cell death than control after galectin-9 treatment (Fig. 3g).

### Lnc-Tim3 induces an exhausted-like phenotype via suppressing Tim-3–Bat3 signaling

**Fig. 4:**
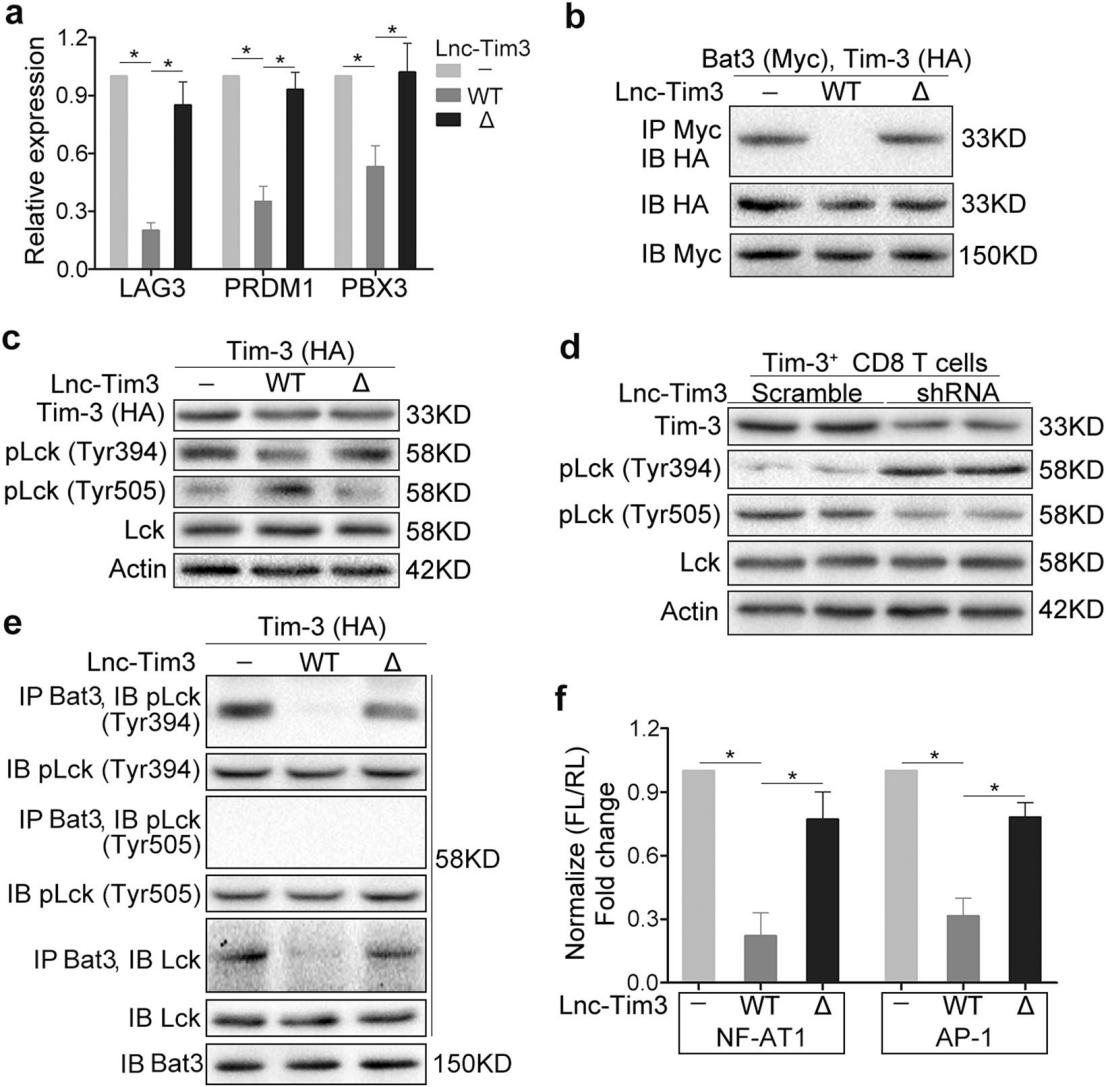
Upregulated Lnc-Tim3 in CD8 T cells induces an exhausted-like phenotype via suppressing Tim-3-Bat3 signaling. **a** CD8 T cells isolated from peripheral blood of healthy human were transfected with indicated lentiviral particles (wild type Tim-3 and Lnc-Tim3 wild type or Δ226-277). The mRNA levels of LAG3, PRDM1, and PBX3 were assessed by real-time PCR after cells treated with anti-TCR/anti-CD28 beads and PMA-ionomycin. (*n* = 10). **b** A western blot of Bat3-Myc, Tim-3–HA, and Lnc-Tim3 (wild type or Δ226-277) overexpressed in HEK 293 T cells and coimmunoprecipitated to confirm the specificity of the Bat3–Tim-3 interaction in mammalian cells. IP, immunoprecipitation; IB, immunoblot. Representative of three experiments. **c** A western blot of pLck (Tyr394 and Tyr505) expression from Jurkat T cells that were transfected with indicated lentiviral particles (Tim-3–HA, Lnc-Tim3 wild type or Δ226-277) and anti-TCR/anti-CD28 beads. Representative of three experiments. (**d**) Lnc-Tim3^high^Tim-3^+^CD8 T cells isolated from tumor-infiltrating lymphocytes (TILs) of HCC patients with Lnc-Tim3 high expression were transfected with indicated lentiviral particles (Lnc-Tim3 scramble or shRNA) and treated with anti-TCR/anti-CD28 beads. The expression of pLck (Tyr394 and Tyr505) was assessed by western blot. Representative of three experiments. **e** Jurkat T cells were transfected with indicated lentiviral particles (Tim-3–HA, Lnc-Tim3 wild type or Δ226-277) and treated with anti-TCR/anti-CD28 beads and PMA-ionomycin, followed by coimmunoprecipitated to confirm the specificity of the Bat3-pLck (Tyr394 and Tyr505) interaction in cells. Representative of three experiments. **f** Jurkat T cells were transfected with indicated lentiviral particles (Tim-3–HA, Lnc-Tim3 wild type or Δ226-277). Then, Jurkat T cells were also transfected with NFAT1 or AP-1 luciferase reporter. NFAT1 or AP-1 luciferase activity was determined after a 6-h stimulation (anti-TCR/anti-CD28 beads and PMA-ionomycin). Data are presented as means ± S.E.M. (*P < 0.05)

To confirm the importance of Lnc-Tim3 for its negative regulatory effects on T cell exhaustion and T cell signaling, we assessed the expression of exhaustion-associated markers of Lnc-Tim3 (wild-type and mutant)-overexpressing Jurkat T cells. Overexpression of Lnc-Tim3_WT_, but not Lnc-Tim3_Δ251-302_, upregulated a number of exhaustion-associated markers, such as the transcription factors LAG3, PRDM1 and PBX3 (Fig. 4a). A later investigation has shown that Bat3 binds to IC domain of Tim-3 and recruits the catalytically active form of Lck (phosphorylated at Tyr394 (pTyr394)), thereby forming an IC molecular complex with Tim-3 that preserves and potentially promotes T cell signaling and represses Tim-3-mediated cell death and exhaustion. To confirm that Lnc-Tim3 is able to inhibit Bat3–Tim-3 interaction via binding to the IC domain of Tim-3, we overexpressed Bat3-Myc and Tim-3-HA constructs in HEK 293 T cells and found that Tim-3 coimmunoprecipitated with Bat3 (Fig. 4b). Using the Lnc-Tim3 deletion constructs Lnc-Tim3_Δ251–302_, we identified residues 251–302 of Lnc-Tim3 as being crucial for mediating inhibiting Bat3–Tim-3 interaction (Fig. 4b). Bat3 recruits the catalytically active form of Lck (pLck Tyr394), thereby forming an IC molecular complex with Tim-3^19^. Further, we observed the levels of pLck Tyr394 and pLck Tyr505 in Jurkat T cell that were lentivirally overexpressed of Lnc-Tim3_WT_ or Lnc-Tim3_Δ251–302_. The results showed that the catalytically active form of Lck (pLck Tyr394) was reduced and a substantial amount of catalytically inactive Lck (pLck Tyr505) accumulated in Lnc-Tim3-WT transfected Jurkat T cells (Fig. 4c). In contrast, Δ251–302 restored the inhibitory effects of Lnc-Tim3 on Lck activation (Fig. 4c). Similarly, the inhibitory effects of Lnc-Tim3 on Lck activation was also confirmed in Tim-3^+^CD8 T cells from TILs of HCC patients (Fig. 4d). Notably, Bat3 could not pull down the catalytically active form of Lck (pTyr394) only in Lnc-Tim3 transfected Jurkat T cells (Fig. 4e). These data indicate that the inhibition effects of Lnc-Tim3 on Tim-3–Bat3 signaling is dependent on the predicted region (251 to 302). The cytoplasmic tail of Tim-3 is critical inducing NFAT1 and AP-1 signaling activation^15^. Interestingly, we observed that wild type Lnc-Tim3 reduced the NFAT1 and AP-1 activation compared to control in Jurkat T cells with anti-TCR/CD28 stimulation. However, the Lnc-Tim3_Δ251–302_ led to a much more dramatic restoration of NFAT1 and AP-1 activation (Fig. 4f).

### Lnc-Tim3 leads to nuclear localization of Bat3 and enhances p300-dependent p53 and RelA transcriptional activation

**Fig. 5:**
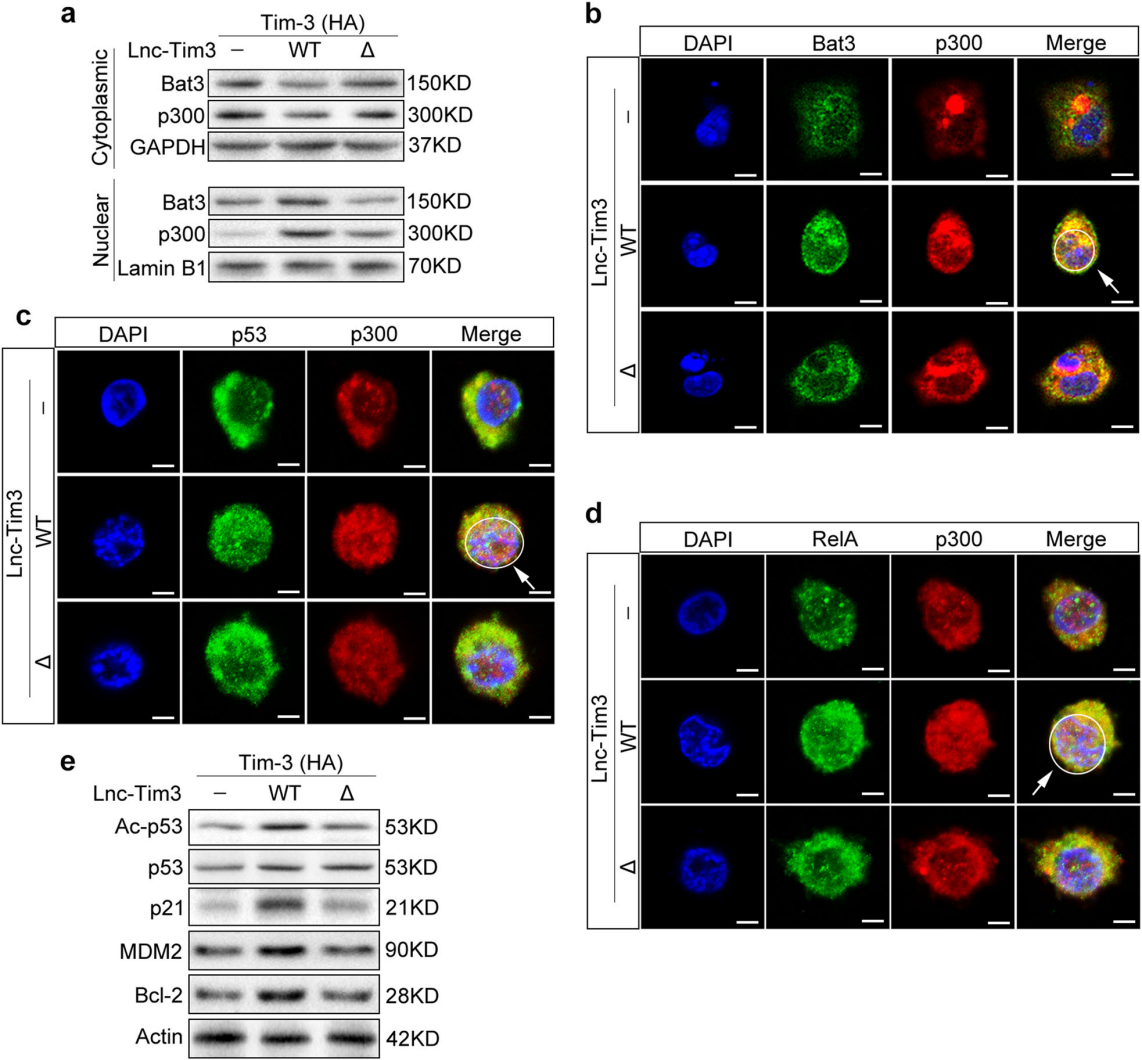
Upregulated Lnc-Tim3 leads to nuclear localization of Bat3 and enhances p300-dependent p53 and RelA transcriptional activity. **a**,** b** Jurkat T cells were transfected with indicated lentiviral particles (Tim-3 wild type, Lnc-Tim3 wild type or Δ226-277) and treated with anti-TCR/anti-CD28 beads and PMA-ionomycin. After 6 h, cytosolic and nuclear fractions were prepared from extracts. Nuclear localization of Bat3 and p300 was determined by western blot (**a**) and confocal scanning laser microscopy (**b**). The white arrow and circle represent Nuclei. Representative of three experiments. **c**,** d** Jurkat T cells were transfected with indicated lentiviral particles (Tim-3 wild type, Lnc-Tim3 wild type or Δ226-277) and treated with anti-TCR/anti-CD28 beads and PMA-ionomycin. Nuclear localization of p300 and p53 (**c**) or RelA (**d**) was determined by confocal scanning laser microscopy. The white arrow and circle represent Nuclei. Representative of three experiments. **e** Jurkat T cells were transfected with indicated lentiviral particles (Tim-3 wild type, Lnc-Tim3 wild type or Δ226-277) and treated with anti-TCR/anti-CD28 beads and PMA-ionomycin. Protein levels of Ac-p53, p21, MDM2, and Bcl-2 were determined by western blot. Representative of three experiments

Bat3 interacts with histone acetyltransferases p300 in the cytoplasm and is responsible for p300 nuclear translocation via the nuclear localization signal at its C terminus^20^. To determine whether the released Bat3 could form a complex with p300 and increase its nuclear translocation, we prepared nuclear and cytosolic fractions from Jurkat T cells that had been transfected with either Lnc-Tim3_WT_ or Lnc-Tim3_Δ251–302_ and Tim-3-HA. Lnc-Tim3_WT_, but not Lnc-Tim3_Δ251–302_, leads to the Bat3 and p300 proteins nuclear translocation (Fig. 5a). To confirm these biochemical findings at the cellular level, we investigated the subcellular localization of Bat3 and p300 in Jurkat T cells. In Lnc-Tim3_WT_ transfected cells, Bat3 and p300 could colocalized in the nucleus (Fig. 5b). However, in Lnc-Tim3_Δ251–302_ transfected cells, Bat3 and p300 was stained only very weakly in the nucleus (Fig. 5b). Bat3 enhances the recruitment of p53 to p300 and facilitates subsequent p53 acetylation^21^. The interaction of the RelA subunit of NF-kB with CBP/p300 is vital for RelA-dependent gene transcription^22^. The subcellular localization of p300 and p53 was assessed by immunofluorescence and we found that p53 was detected in nuclei and colocalized with p300 after transfection with Lnc-Tim3_WT_. In contrast, Δ251–302 of Lnc-Tim3 did result in colocalization of p53 and p300 (Fig. 5c). Similarly, Lnc-Tim3_WT_, but not Lnc-Tim3_Δ251–302_, promoted the colocalization of RelA and p300 in the nuclei (Fig. 5d). Furthermore, we found that Lnc-Tim3 increased p53 acetylation and the expression of p21, MDM2, and Bcl-2. However, deletions predicted region (251–302) of Lnc-Tim3 abrogated the transcriptional activation of these genes (Fig. 5e). These results suggested that upregulated Lnc-Tim3 could promote expression of anti-apoptosis proteins, thereby contributing to the survival of Tim-3^+^ exhausted CD8 T cells.

## Discussion

Immunotherapy is an attractive choice to decrease the risk of relapse through eliminating micrometastatic residual disease after surgical or percutaneous ablation in HCC^3,23^. Immune checkpoint inhibitors, such as ipilimumab (anti-CTLA-4), nivolumab (anti-PD-1) and pembrolizumab (anti-PD-1), which have already received regulatory approval^24,25^. Furthermore, several properties of Tim-3 make it a perfect target for the next generation of immunotherapy. For example, its selective expression on intratumoral T cells may allow for more precise therapy via the targeting of tumor-infiltrating T cells, potentially reducing non-specific toxicity^26^. However, the underlying mechanisms are not well understood and seemingly even contradictory to well-established T cell-stimulating function of Tim-3^27^.

LncRNAs exhibit dynamic expression in cell-type, developmental-stage and context-specific manners to coordinate several aspects of immune function in the immune system^28^. Therefore, it is critical for us to know more about lncRNAs biological functions and elucidate the mechanism by which lncRNAs exert their immunoregulatory effects. LncRNA-CD244 recruits the EZH2 complex to IFN-γ and TNF genes promoter, thus suppressing the expression of IFN-γ and TNF-α in CD8 T cells^29^. Linc-CCR2-59AS significantly promotes the migration of effector Th2 cells into the lung of C57BL/6 mice^30^. In our previous study, we have extracted T cells from both blood and the tissues of three patients with HCC and the blood of three healthy volunteers. The lncRNAs and mRNAs expression profiles of CD3 T cells has been established. Among them, Lnc-EGFR promotes HCC immune evasion via stimulating T-regulatory cells differentiation^17^. In this study, we focus on the dysregulated lncRNAs and related lncRNA-mRNA co-expression networks in tumor-infiltrating CD8 T cells. We found that Lnc-Tim3 (ENST00000443947.1) was upregulated and negatively correlated with the percentage of IFN-γ^+^ CD8 T cells in the tumor-infiltrating CD8 T cells of HCC patients.

T cell exhaustion is identified as a natural mechanism for restricting immune pathology, although it may be desirable to circumvent this mechanism to help eliminate tumors^31^. Up to date, transmembrane proteins PD-1, LAG3, and Tim-3 are considered as the markers of exhausted T cells^32^. However, Tim-3 also has been found to actually increase signaling downstream of TCR/CD3, at least under acute conditions^33^. Interestingly, we found that upregulated Lnc-Tim3 was correlated with the exhaustion of CD8 T lymphocytes. It has been verified that Bat3 binds to the IC domain in the C-terminal tail of Tim-3, thus recruiting the catalytically active form of Lck (pLck Tyr505), promoting T cell signaling activation, and repressing CD8 T cell exhaustion^19^. In our study, the results show that Lnc-Tim3 specifically binds to the IC domain of Tim-3. Tim-3 leads to release of Bat3 from the Tim-3 tail, thereby promoting Tim-3-mediated T cell inhibition by accumulating catalytically inactive form of Lck (pLck Tyr505). Bat3-overexpressing Th1 cells produced greater amounts of IFN-γ and IL-2 and significantly more IFN-γ^19^. Similarly, our study demonstrates that Lnc-Tim3_WT_, but not Lnc-Tim3_Δ251–302_, strikingly reduced the amounts of IFN-γ and IL-2, the percentage of IFN-γ secreting CD8 T cells, and IFN-γ secretion. Src family tyrosine kinase Lck has two different forms, inactivated form (pLck Tyr505) and activated form (pLck Tyr394)^34^. The engagement of the TCR and coreceptor is critical for T-cell immune response initiation. Activation of Lck (pLck Tyr394) is central to the initiation of TCR signaling pathways^35^. Activated Lck phosphorylates ZAP-70 that, in turn, activates the transcription factors NFAT and AP-1, thereby driving T-cell immune response to antigen^36^. Interestingly, we observed that wild type Lnc-Tim3 reduced the activation of transfactor NFAT1 and AP-1. However, deletions predicted region (251–302) of Lnc-Tim3 led to a much more dramatic restoration of NFAT1 and AP-1 activation. These results suggest that Lnc-Tim3 induces an exhausted-like phenotype via suppressing Tim-3–Bat3 signaling and downstream signaling pathway NFAT1 and AP-1 in CD8 T cells.

It has been studies that Tim-3 causes a replicative senescent of CD8 lymphocytes from HCC TILs. Bat3 interacts with histone acetyltransferases p300 in the cytoplasm and is responsible for p300 nuclear translocation via the nuclear localization signal at its C terminus^20,21^. According to the results, we discover that the interactions between Lnc-Tim3 and Tim-3 leads to release of Bat3 from the Tim-3 IC tail and the released Bat3 is then free to form a complex with p300, thereby increasing its nuclear translocation. Nuclear Bat3 enhances the recruitment of p53 to p300 and facilitates subsequent acetylation of p53 and transcription of p21. The acetylation of p53 modulates p21-dependent cell-cycle arrest^37,38^. It was revealed that interaction between p53 and p21 could inhibit interaction of cyclin-A/CDK2 complex with blocking the cell cycle through G1/S^39,40^. These results suggest that Lnc-Tim3 may induce the replicative senescent of CD8 T cells in HCC TILs via promoting the formation of p300/p53/p21 complex.

In HCC, IFN-γ derived from TILs induced Gal-9 expression by Kupffer cells and Tim-3^+^ T cells co-localized with Gal-9^+^ Kupffer cells within the tumor^41^. However, Tim-3^+^ exhausted CD8 T cells persist in HCC TILs^6^. Interestingly, upregulated Lnc-Tim3 could promote expression of anti-apoptosis proteins, thereby contributing to the survival of Tim-3^+^ exhausted CD8 T cells in HCC. In our study, Bat3 enhances the recruitment of RelA to p300 and facilitates subsequent transcription of MDM2 and Bcl-2. RelA has an important role in inflammatory response and T cell survival through regulating the activation of related-genes^42^. Furthermore, CBP/p300 can recruit RelA to its target promoter sites^22^. Additionally, RelA binds to the κB2 site in the MDM2 P1 promoter and induces MDM2 transcriptional activation. MDM2 blocks Bim-dependent apoptosis via binding and inhibition of p73, thus contributing to the survival of activated T cells^43^. Furthermore, RelA promotes the survival of T cells also by upregulating the anti-apoptotic protein Bcl-2^44,45^.

**Fig. 6:**
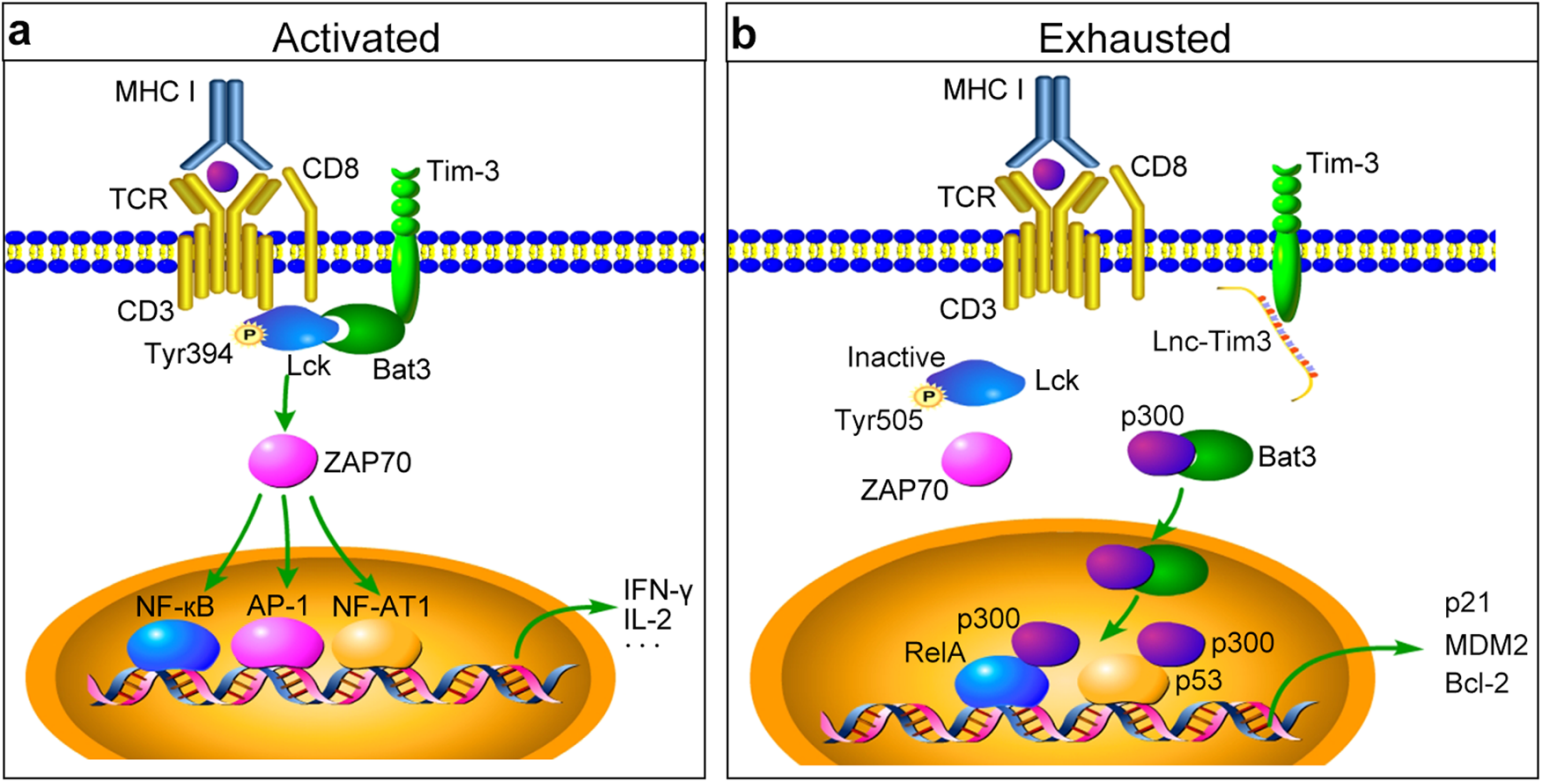
Long non-coding RNA Lnc-Tim3 exacerbates CD8 T cell exhaustion by binding to Tim-3 and inducing nuclear translocation of Bat3 in HCC. **a** Briefly, Bat3 can bind to the intracellular domain in the C-terminal tail of Tim-3. Bat3 recruits the catalytically active form of Lck (pLck Tyr394), thereby forming an intracellular molecular complex with Tim-3 that preserves and potentially promotes T cell signaling (ZAP70/AP-1/NFAT1), endogenous cytokine production (IL-2/IFN-γ), and represses CD8 T cell exhaustion. **b** Interactions between long non-coding RNA Lnc-Tim3 and Tim-3 leads to release of Bat3 from the Tim-3 tail, thereby promoting Tim-3-mediated T cell inhibition by accumulating catalytically inactive form of Lck (pLck Tyr505). Additionally, the released Bat3 is then free to form a complex with p300 and increases its nuclear translocation. Bat3 enhances the recruitment of p53 and RelA to p300 and facilitates subsequent acetylation of p53 and transcription of p21, MDM2, and Bcl-2

In summary, we have revealed that Lnc-Tim3 links CD8 T cells exhaustion and HCC. Lnc-Tim3 specifically binds to the IC tail of Tim-3, thereby leading to release of Bat3 from the Tim-3 tail, accumulating catalytically inactive form of Lck (pLck Tyr505), suppressing downstream signaling pathway NFAT1 and AP-1 in CD8 T cells. Additionally, the released Bat3 forms a complex with p300 and increases its nuclear translocation, leading to recruitment of p53 and RelA to p300, promoting cell cycle arrest, and protecting from death in CD8 T cells (Fig. 6). These data imply an important role of Lnc-Tim3 in inducing CD8 T cells exhaustion, a phenotype which is correlated with compromised anti-tumor immunity. Since Tim-3 is considered to be an ideal target for the next generation of immunotherapy, these findings might suggest that Lnc-Tim3 and its associated signaling pathways may influence the outcome of cancer therapies aimed at modulating the acquired immune system.

## Materials and Methods

### Clinical samples

Blood or tissue samples from 40 healthy volunteers and 55 HCC patients who received treatment between May 2014 and May 2016 at The First Affiliated Hospital of Nanjing Medical University (Nanjing, Jiangsu, China) were used for isolation of peripheral or tissue-infiltration lymphocytes. None of the patients had received anticancer therapy before surgery, and individuals with concurrent autoimmune disease, HIV, or syphilis were excluded. Clinical characteristics were classified according to the guidelines of Union for International Cancer Control (UICC TNM). All experiments were performed in compliance with government policies and the Helsinki Declaration. The individuals were informed about the study and gave consent prior to the specimen collection. And the research has been approved by an ethics committee of the First Affiliated Hospital of Nanjing Medical University.

### Statistical analysis

Data are presented as mean ± S.E.M. The Student’s t-test and analysis of variances were used to evaluate statistical differences in clinical characteristics. All the expression experiments we conducted in vitro were repeated at least three times with samples in triplicates. Pearson correlation analysis was used to analyze the relationship of associated factors. Statistical analysis was performed using STATA 9.2 and presented with the GraphPad prism software (CA, USA). In all cases, *P* < 0.05 was considered significant.

Details materials and methods were described in Supplementary materials and methods. Supplementary information is available at Cell Death and Disease website (https://www.nature.com/cddis).

## Electronic supplementary material

Supplementary Information
