# Supplementary material for: Long non-coding RNA Lnc-Tim3 exacerbates CD8 T cell exhaustion via binding to Tim-3 and inducing nuclear translocation of Bat3 in HCC
Source: Cell Death Dis. 2018 Apr 30;9(5):478. doi: 10.1038/s41419-018-0528-7 (PMC5924754; doi:10.1038/s41419-018-0528-7)
Supplement: Supplementary file 1 — Supplementary Information [file 41419_2018_528_MOESM1_ESM.docx]

**Supplementary Information for:**

**Long noncoding RNA Lnc-Tim3 exacerbates CD8 T cell exhaustion via binding to Tim-3 and inducing nuclear translocation of Bat3 in HCC**

Jie Ji^1,§^, Yin Yin^1,§^, Huanyu Ju^2,§^, Xiaoliang Xu^1^, Wei Liu^3^, Qiang Fu^3^, Jiaojiao Hu^3^, Beicheng Sun^1,*^

1. Liver Transplantation Center of the First Affiliated Hospital and State Key Laboratory of Reproductive Medicine, Nanjing Medical University, Nanjing, Jiangsu Province, P.R.China.

2. Department of General Surgery, The Second Affiliated Hospital of Nanjing Medical University, Nanjing, Jiangsu Province, P.R.China.

3. Department of Gastroenterology, The Second Affiliated Hospital of Nanjing Medical University, Nanjing, Jiangsu Province, P.R.China.

^§^: The authors contributed equally to this work.

*Corresponding authors:

Dr. Beicheng Sun, Liver Transplantation Center, The First Affiliated Hospital of Nanjing Medical University, 300 Guangzhou Road, Nanjing, Jiangsu Province, P.R. China.

Phone: 86-25-68136746, Fax: 86-25-86560946

E-mail: sunbc@njmu.edu.cn

**Contents:**

**1.** **Supplementary materials and methods**

***Clinical samples.***

Blood or tissue samples from 40 healthy volunteers and 55 HCC patients who received treatment between May 2014 and May 2016 at The First Affiliated Hospital of Nanjing Medical University (Nanjing, Jiangsu, China) were used for isolation of peripheral or tissue-infiltration lymphocytes. None of the patients had received anticancer therapy before surgery, and individuals with concurrent autoimmune disease, HIV, or syphilis were excluded. Clinical characteristics were classified according to the guidelines of Union for International Cancer Control (UICC TNM). All experiments were performed in compliance with government policies and the Helsinki Declaration. The individuals were informed about the study and gave consent prior to the specimen collection. And the research has been approved by an ethics committee of the First Affiliated Hospital of Nanjing Medical University.

***Cell culture***

Human Jurkat T cells were maintained in RPMI-1640 medium (Invitrogen Life Technologies, CA, USA) supplemented with 10% fetal bovine serum (FBS, Gibco, CA, USA). Jurkat T cell line was purchased from cell bank of Chinese Science Academy. 293 T cells obtained from ATCC were cultured in DMEM supplemented with 10% FBS. All cell lines were tested for mycoplasma contamination.

***Mutagenesis of Lnc-Tim3 and Tim-3 and lentiviral packaging.***

The full-length of Bat3 (myc), the full-length of and mutants of Lnc-Tim3 (ENST00000443947.1) and Tim-3 (HA) were synthesized by Genscript Co. Ltd. (Nanjing, China) based on the sequence indicated in Supplementary Table 1. Then the sequences were subcloned into pLV plasmid, and further packaged for lentivrial particles according to the method as previously described ^1^. In brief, candidate plasmid was co-transfected with VSV-G and dR8.91 in 293 T cell line. The supernatant was collected after culturing for 72 h. Virus supernatant was concentrated through ultracentrifugation.

***Lymphocyte isolation and culture***

PBLs were isolated by Ficoll (BD Pharmingen, CA, USA) density gradient centrifugation according to the method as previously described. Fresh TILs were obtained as described previously ^1^. Briefly, liver cancer tissue specimens were cut into small pieces and digested in RPMI-1640 medium. Dissociated cells were filtered through a 75 mm cell strainer and separated by Ficoll centrifugation, and the mononuclear cells were washed and resuspended in RPMI-1640 supplemented with 10% FBS. T cells were purified with anti-CD3 magnetic Dynabeads (Invitrogen, USA) according to manufacturer’s instruction. Indicated viral particles were concentrated by ultracentrifugation and expression vector titres were determined. The CD8 T cells isolated from HCC patients were cultured with TAKARA GT-T551 medium (TAKARA, Japan) supplied with human IL-2, and then transduced with lentivirus with desired expression vectors.

***RNA isolation and real-time PCR***

Total RNA was isolated with Trizol and purified with the RNeasy MinElute Clean up kit (Qiagen, Hilden, Germany) according to the manufacturer’s instruction. The cDNA was synthesized from the total RNA using the random priming method according to the method as previously described ^1^. Transcript levels were measured in duplicate by real-time PCR (ABI 7900, Life Technologies). Expression levels were calculated relative to GAPDH. Primer pairs used in SYBR Green reactions are listed in Supplementary Table 1.

***Immunofluorescence analysis***

The immunofluorescence analysis was performed as previously described 9, cells were stained with rabbit anti-human p300, mouse anti-human Bat3, mouse anti-human p53, and mouse anti-human IFN-g antibodies, followed by staining with Alexa Fluor 488-conjugated anti-mouse IgG (1:500, Ab150117) and Alexa Fluor 594-conjugated anti-rabbit IgG (1:1,000, Ab150080) (Abcam, Cambridge, UK) antibodies. The results were detected by confocal microscopy (Zeiss, Oberkochen, Germany).

***In vitro stimulation of CD8 T cells and Flow cytometry (FCM)***

Magnetic beads isolated peripheral T lymphocytes, tumour-infiltrating T lymphocytes, and various genes modified T cells were expanded with Dynabeads Human T-Activator CD3/CD28 (ThermoFisher, CA, USA). For detection of intracellular cytokines, CD8 T cells were stimulated at 37 ºC for 5 h with Leukocyte Activation Cocktail (BD Pharmingen, USA) and anti-TCR antibody. AFP158-166, GPC3144-152, and NY-ESO-1157-165 were synthesized by Genscript Co. Ltd. (Nanjing, China). Antibody to the Jurkat TCR was purified from the C305.2 hybridoma, which was obtained from ATCC (CRL-2424, Manassas, VA, USA). Thereafter, cells were, fixed, permeabilized with IntraPrep reagent (BD Pharmingen, USA), and then stained with flurochrome-conjugated labelled antibodies including Tim-3 (565570), CD8 (557085), IL-2 (554567), and IFN-γ (57995) and 7-AAD (559925) (BD Pharmingen). Data were acquired on BD FACSVerse flow cytometer (BD Pharmingen, USA).

***RNA pull-down and mass spectrometry***

RNA pull-down was performed according to the method as previously described ^1^. The biotin-labelled lncRNA (both wild type and mutant type) and the antisense RNA were in vitro transcribed with a Biotin RNA Labelling Mix (Roche, CA, USA) and the T7 RNA polymerase (Roche), treated with RNase-free DNase I (Roche) and purified with an RNeasy Mini Kit (Qiagen). CD 8 T cell extracts were incubated with biotinylated RNAs and 60 ml of streptavidin agarose beads (Invitrogen Life Technologies). The associated proteins were resolved by SDS–polyacrylamide gel electrophoresis, and specific bands were excised. Proteins were eluted, digested and subjected to the OrbitrapVelos Pro LC/MS system (Thermo Scientific, CA, USA). Data were analysed by Proteome Discoverer and the resulting peak lists were used for searching the NCBI protein database with the Mascot search engine.

***RNA Immunoprecipitation***

RIP was carried out by using the Magna RIP RNA-Binding Protein Immunoprecipitation Kit (Millipore, MA, USA) according to the method as previously described ^1^. Anti-HA (Tim-3) antibodies (1:50, ab9110; Abcam, Cambridge, UK) were used for RIP, respectively. CD8 T cells were either transduced with fixed or different doses of lentivirus containing Lnc-Tim3 along with other indicated virus. The co-precipitated RNAs were detected by reverse transcription PCR and real-time PCR. The primer sequences are listed in Supplementary Table 1. Total RNAs (input controls) and IgG were assayed simultaneously to demonstrate that the detected signals were the result of RNAs specifically binding to Tim-3.

***Immunoprecipitation and western blot***

The whole-cell lysates were prepared as previously described ^1^. Equal amounts of proteins were boiled, separated on 10% SDS–polyacrylamide gel electrophoresis, transferred onto a PVDF membrane and visualized via an ECL kit (Millipore, MA, USA). Antibodies including Myc tag (1:1,000, ab9106), Lck (1:1,000, ab3885), p-Lck (Y394) (1:1,000, ab208787), p-Lck (Y505) (1:1,000, ab4901), Bat3 (1:1,000, ab137076), p300 (1:1,000, ab54984), p53 (acetyl K373) (1:1,000, ab62376), p53 (1:1,000, ab26), p21 (1:1,000, ab109520), MDM2 (1:1,000, ab38618), Bcl-2 (1:1,000, ab32124), Lamin B1 (1:1,000, ab 16048), GAPDH (1:1,000, ab8245), and actin (1:1,000, ab8226) were purchased from Abcam (Cambridge, UK).

***Dual-luciferase reporter assay***

Jurkat T cells were transfected with a luciferase reporter construct with multiple NFAT1 (TTCC) or AP-1 (TGACTAA) response elements. The NFAT1 or AP-1 response elements luciferase reporter vectors (pGL4 packaged) and the mock pGL4 vector were electroporated into Jurkat T cells. After 24 h, cells were starved in serum-free medium and stimulated with Dynabeads Human T-Activator CD3/CD28 (ThermoFisher, CA, USA), and luciferase activity was measured by the dual luciferase assay system (Promega, WI, USA) according to the manufacturer’s instructions. Data were normalized by the activity of Renilla luciferase.

***Statistical analysis***

Data are presented as mean ± S.E.M. The Student’s t-test and analysis of variances were used to evaluate statistical differences in clinical characteristics. All the expression experiments we conducted in vitro were repeated at least three times with samples in triplicates. Pearson correlation analysis was used to analyze the relationship of associated factors. Statistical analysis was performed using STATA 9.2 and presented with the GraphPad prism software (CA, USA). In all cases, *P* < 0.05 was considered significant.

Details materials and methods were described in Supplementary materials and methods. Supplementary information is available at Cell Death and Disease website (http://www.nature.com/cddis)

***Data availability statement***

Microarray detection, screening workflow, and co-expression network analysis prepared as previously described ^1^. The Lnc-Tim3/Tim-3-binding site sequence was provided by the CatRAPID database. The microarray data have been deposited in the ArrayExpress (https://www.ebi.ac.uk) database under the accession code E-MTAB-3553. The microarray data referenced during the study are available in a public repository from the ArrayExpress website (https://www.ebi.ac.uk). All the other data supporting the findings of this study are available within the article and its Supplementary Information files or from the corresponding author upon reasonable request.

***Detailed sequence information for mutation construction and primer***

**Lnc-Tim3 (ENST00000443947.1),**

**Wild type,**

AGATGCTAAGCCTGGAAACCCACAGGAAACATGTTATTCTTCAGTATTTGCAAAGCACTGATAAAAACATCTCTCTTCTCTCAATCTTGCTTCCTTCTTTTCATTTACTAAATAGGCTTTTGAGCCTCACAAGACTTTGAGATTTTATGGATATGAAACCTGCTATCATTTAGCATCCTGCCTCTGGGAATAGAAGCTGGACTGTACTGCTGCCATCTCGGCTCACTGCAACCTCCCTGCCTGATTCTCCTGCCTCAGCCTGCCCAGTGCCTGCGATTGCAGGCGCGCGTCGCCACGCCTGACTGGTTTTCATATTTTTTGGGTGGAGACGGGGTTTCCCTGTGTTGGCCGGGCTGGTCTCCAGCTCCTAACCGCGAGTGATCCGCCAGCCTCCGCCTCCCGAGGTGCCCGGATTGCAGACGGAGTCTCCTTCACTCAGTGCTCAATGGTGCCCAGGCTGGAGTGCAGTGGTGTGATCTCGGCTCGCTACAACATCCACCTCCCAGCAGCCTGCCTTGGCCTCCCAAAGTGCCGAGATTGCAGCCTCTGCCCGGCCGCCACCCCGTCTGGGAAGT

**Mutant (Δ251-302)**

AGATGCTAAGCCTGGAAACCCACAGGAAACATGTTATTCTTCAGTATTTGCAAAGCACTGATAAAAACATCTCTCTTCTCTCAATCTTGCTTCCTTCTTTTCATTTACTAAATAGGCTTTTGAGCCTCACAAGACTTTGAGATTTTATGGATATGAAACCTGCTATCATTTAGCATCCTGCCTCTGGGAATAGAAGCTGGACTGTACTGCTGCCATCTCGGCTCACTGCAACCTCCCTGCCTGATTCTCCCTGGTTTTCATATTTTTTGGGTGGAGACGGGGTTTCCCTGTGTTGGCCGGGCTGGTCTCCAGCTCCTAACCGCGAGTGATCCGCCAGCCTCCGCCTCCCGAGGTGCCCGGATTGCAGACGGAGTCTCCTTCACTCAGTGCTCAATGGTGCCCAGGCTGGAGTGCAGTGGTGTGATCTCGGCTCGCTACAACATCCACCTCCCAGCAGCCTGCCTTGGCCTCCCAAAGTGCCGAGATTGCAGCCTCTGCCCGGCCGCCACCCCGTCTGGGAAGT

**Tim-3, Gene ID: 84868,**

**Wild type,**

ATGTTTTCACATCTTCCCTTTGACTGTGTCCTGCTGCTGCTGCTGCTACTACTTACAAGGTCCTCAGAAGTGGAATACAGAGCGGAGGTCGGTCAGAATGCCTATCTGCCCTGCTTCTACACCCCAGCCGCCCCAGGGAACCTCGTGCCCGTCTGCTGGGGCAAAGGAGCCTGTCCTGTGTTTGAATGTGGCAACGTGGTGCTCAGGACTGATGAAAGGGATGTGAATTATTGGACATCCAGATACTGGCTAAATGGGGATTTCCGCAAAGGAGATGTGTCCCTGACCATAGAGAATGTGACTCTAGCAGACAGTGGGATCTACTGCTGCCGGATCCAAATCCCAGGCATAATGAATGATGAAAAATTTAACCTGAAGTTGGTCATCAAACCAGCCAAGGTCACCCCTGCACCGACTCGGCAGAGAGACTTCACTGCAGCCTTTCCAAGGATGCTTACCACCAGGGGACATGGCCCAGCAGAGACACAGACACTGGGGAGCCTCCCTGATATAAATCTAACACAAATATCCACATTGGCCAATGAGTTACGGGACTCTAGATTGGCCAATGACTTACGGGACTCTGGAGCAACCATCAGAATAGGCATCTACATCGGAGCAGGGATCTGTGCTGGGCTGGCTCTGGCTCTTATCTTCGGCGCTTTAATTTTCAAATGGTATTCTCATAGCAAAGAGAAGATACAGAATTTAAGCCTCATCTCTTTGGCCAACCTCCCTCCCTCAGGATTGGCAAATGCAGTAGCAGAGGGAATTCGCTCAGAAGAAAACATCTATACCATTGAAGAGAACGTATATGAAGTGGAGGAGCCCAATGAGTATTATTGCTATGTCAGCAGCAGGCAGCAACCCTCACAACCTTTGGGTTGTCGCTTTGCAATGCCATAG

**Mutant (Δ226-277)**

ATGTTTTCACATCTTCCCTTTGACTGTGTCCTGCTGCTGCTGCTGCTACTACTTACAAGGTCCTCAGAAGTGGAATACAGAGCGGAGGTCGGTCAGAATGCCTATCTGCCCTGCTTCTACACCCCAGCCGCCCCAGGGAACCTCGTGCCCGTCTGCTGGGGCAAAGGAGCCTGTCCTGTGTTTGAATGTGGCAACGTGGTGCTCAGGACTGATGAAAGGGATGTGAATTATTGGACATCCAGATACTGGCTAAATGGGGATTTCCGCAAAGGAGATGTGTCCCTGACCATAGAGAATGTGACTCTAGCAGACAGTGGGATCTACTGCTGCCGGATCCAAATCCCAGGCATAATGAATGATGAAAAATTTAACCTGAAGTTGGTCATCAAACCAGCCAAGGTCACCCCTGCACCGACTCGGCAGAGAGACTTCACTGCAGCCTTTCCAAGGATGCTTACCACCAGGGGACATGGCCCAGCAGAGACACAGACACTGGGGAGCCTCCCTGATATAAATCTAACACAAATATCCACATTGGCCAATGAGTTACGGGACTCTAGATTGGCCAATGACTTACGGGACTCTGGAGCAACCATCAGAATAGGCATCTACATCGGAGCAGGGATCTGTGCTGGGCTGGCTCTGGCTCTTATCTTCGGCGCTTTAATTTTCAAATGGAATGAGTATTATTGCTATGTCAGCAGCAGGCAGCAACCCTCACAACCTTTGGGTTGTCGCTTTGCAATGCCATAG

**Bat3, Gene ID: 7917,**

ATGGAGCCTAATGATAGTACCAGTACCGCTGTGGAGGAGCCTGACAGCTTGGAGGTGTTGGTGAAGACCTTGGACTCTCAAACTCGTACCTTTATTGTGGGGGCCCAGATGAATGTAAAAGAGTTTAAGGAGCACATTGCTGCCTCTGTCAGCATCCCATCTGAAAAACAACGGCTCATTTACCAGGGACGAGTTCTGCAAGATGATAAGAAGCTTCAGGAATACAATGTTGGGGGAAAGGTTATCCACCTGGTGGAACGGGCTCCTCCTCAGACTCACCTCCCTTCTGGGGCATCTTCTGGGACGGGGTCTGCCTCAGCCACTCATGGTGGGGGATCCCCCCCTGGTACTCGGGGGCCTGGGGCCTCTGTTCATGACCGGAATGCCAACAGCTATGTCATGGTTGGAACCTTCAATCTTCCTAGTGACGGCTCTGCTGTGGATGTTCACATCAACATGGAACAGGCCCCGATTCAGAGTGAGCCCCGGGTACGGCTGGTGATGGCTCAGCACATGATCAGGGATATACAGACCTTACTATCCCGGATGGAGTGTCGAGGAGGGCCCCAACCGCAGCACAGTCAGCCGCCCCCGCAGCCACCGGCTGTGACCCCGGAGCCAGTAGCCTTGAGCTCTCAAACATCAGAACCAGTTGAAAGTGAAGCACCTCCCCGGGAGCCCATGGAGGCAGAAGAAGTGGAGGAGCGTGCCCCAGCCCAGAACCCGGAGCTCACTCCTGGCCCAGCCCCAGCGGGCCCAACACCTGCCCCGGAAACAAATGCACCCAACCATCCTTCCCCTGCGGAGTATGTCGAGGTGCTCCAGGAGCTACAGCGGCTGGAGAGTCGCCTCCAGCCCTTCTTGCAGCGCTACTACGAGGTTCTGGGTGCTGCTGCCACCACGGACTACAATAACAATCACGAGGGCCGGGAGGAGGATCAGCGGTTGATCAACTTGGTAGGGGAGAGCCTGCGACTGCTGGGCAACACCTTTGTTGCACTGTCTGACCTGCGCTGCAATCTGGCCTGCACGCCCCCACGACACCTGCATGTGGTCCGGCCTATGTCTCACTACACCACCCCCATGGTGCTCCAGCAGGCAGCCATTCCCATACAGATCAATGTGGGAACCACTGTGACCATGACAGGAAATGGGACTCGGCCCCCCCCAACTCCCAATGCAGAGGCACCTCCCCCTGGTCCTGGGCAGGCCTCATCCGTGGCTCCGTCTTCTACCAATGTCGAGTCCTCAGCTGAGGGGGCTCCCCCGCCAGGTCCAGCTCCCCCGCCAGCCACCAGCCACCCGAGGGTCATCCGGATTTCCCACCAGAGTGTGGAACCCGTGGTCATGATGCACATGAACATTCAAGATTCTGGCACACAGCCTGGTGGTGTTCCGAGTGCTCCCACTGGCCCCCTGGGACCCCCTGGTCATGGCCAAACCCTGGGACAGCAGGTGCCAGGCTTCCCAACAGCTCCAACCCGGGTGGTGATTGCCCGGCCCACTCCTCCACAGGCTCGGCCTTCCCATCCTGGAGGGCCCCCAGTCTCTGGGACACTGCAGGGCGCCGGTCTGGGTACCAATGCCTCGTTGGCCCAGATGGTGAGCGGCCTTGTGGGGCAGCTTCTTATGCAGCCAGTCCTTGTGGCTCAGGGGACCCCAGGTATGGCTCCACCGCCAGCCCCTGCCACTGCTTCTGCCAGTGCTGGCACCACCAACACAGCTACCACAGCTGGCCCCGCTCCTGGGGGGCCTGCCCAGCCTCCACCCACCCCTCAACCCTCCATGGCTGATCTTCAGTTCTCTCAGCTTCTGGGGAACCTGCTAGGGCCTGCAGGGCCAGGGGCTGGAGGGTCTGGTGTGGCTTCTCCCACCATCACTGTGGCGATGCCTGGTGTCCCTGCCTTTCTCCAAGGCATGACTGACTTCTTGCAGGCAACACAGACAGCCCCTCCACCACCCCCACCTCCTCCACCCCCACCACCTGCCCCAGAGCAGCAGACCATGCCCCCACCAGGCTCCCCTTCTGGTGGCGCAGGGAGTCCTGGAGGCCTGGGTCTTGAGAGCCTGTCACCGGAGTTTTTTACCTCAGTGGTGCAGGGTGTGCTCAGCTCCCTGCTGGGCTCCCTGGGGGCTCGGGCTGGCAGCAGTGAAAGTATTGCTGCCTTCATACAACGCCTCAGTGGATCCAGCAACATCTTTGAGCCTGGAGCTGATGGGGCCCTTGGATTCTTTGGGGCCTTGCTTTCTCTTCTGTGCCAGAACTTCTCTATGGTGGACGTAGTGATGCTTCTCCATGGGCATTTCCAGCCACTACAACGGCTCCAGCCCCAGCTGCGATCCTTCTTCCACCAGCACTACCTGGGTGGTCAGGAGCCCACACCCAGTAACATCCGGATGGCAACCCACACATTGATCACGGGGCTAGAAGAGTATGTGCGGGAGAGTTTTTCCTTGGTGCAGGTTCAGCCAGGTGTGGACATCATCCGGACAAACCTGGAATTTCTCCAAGAGCAGTTTAATAGCATTGCTGCGCATGTGCTGCATTGCACAGATAGTGGATTTGGGGCCCGGTTGCTGGAGTTGTGTAACCAAGGCCTGTTTGAATGCCTGGCCCTAAACCTGCACTGCTTGGGGGGACAGCAGATGGAGCTTGCTGCTGTTATCAATGGCCGAATTCGTCGTATGTCTCGTGGGGTGAATCCCTCCTTGGTGAGCTGGCTGACCACTATGATGGGACTGAGGCTTCAGGTGGTACTGGAGCACATGCCTGTAGGCCCTGATGCCATTCTCAGATACGTTCGCAGGGTTGGTGATCCCCCCCAGCCACTTCCTGAGGAGCCAATGGAAGTTCAGGGAGCAGAAAGAGCTTCCCCTGAGCCTCAGCGGGAGAATGCTTCCCCAGCCCCTGGAACAACAGCAGAAGAGGCCATGTCCCGAGGTCCACCTCCTGCTCCTGAGGGGGGCTCCCGGGATGAACAGGATGGAGCTTCAGCTGAGACAGAACCTTGGGCAGCTGCAGTCCCCCCAGAATGGGTCCCTATTATCCAGCAGGACATTCAGAGCCAGCGGAAGGTGAAACCGCAGCCCCCTCTGAGTGATGCCTACCTCAGTGGTATGCCTGCCAAGAGACGCAAGACGATGCAGGGTGAGGGCCCCCAGCTGCTTCTCTCAGAGGCTGTGAGCCGGGCAGCTAAGGCAGCCGGAGCTCGGCCCCTGACGAGCCCCGAGAGCCTGAGCCGGGACCTGGAGGCACCAGAGGTTCAGGAGAGCTACAGGCAGCAGCTCCGGTCTGATATACAAAAACGACTGCAGGAAGACCCCAACTACAGTCCCCAGCGCTTCCCCAATGCCCAGCGGGCCTTTGCTGATGATCCTTAG

***Primer sequences:***

**Tim-3**

Forward Primer, AAGACCTTGGACTCTCAAACTCG

Reverse Primer, CCTGGTAAATGAGCCGTTGTTTT

**GAPDH**

Forward primer, GTGACTAACCCTGCGCTCC

Reverse primer, CGGTGCCATGGAATTTGCC

**Lnc-Tim3**

Forward primer, TTGGGTGGAGACGGGGTTT

Reverse primer, GGGAGGTGGATGTTGTAGCG

**LAG3**

Forward Primer, GCGGGGACTTCTCGCTATG

Reverse Primer, GGCTCTGAGAGATCCTGGGG

**PRDM1**

Forward Primer, AAGCAACTGGATGCGCTATGT

Reverse Primer, GGGATGGGCTTAATGGTGTAGAA

**PBX3**

Forward Primer, ATTACAGAGCCAAATTGACCCAG

Reverse Primer, TCTCGGAGAAGGTTCATCACAT

1. Jiang R, Tang J, Chen Y, Deng L, Ji J, Xie Y, Wang K, et al. The long noncoding RNA lnc-EGFR stimulates T-regulatory cells differentiation thus promoting hepatocellular carcinoma immune evasion. Nat Commun 2017;8:15129.
